# Supplementary figures and images for: Maintenance of cell wall remodeling and vesicle production are connected in Mycobacterium tuberculosis
Source: eLife. 2025 Feb 17;13:RP94982. doi: 10.7554/eLife.94982 (PMC11832169; doi:10.7554/eLife.94982)

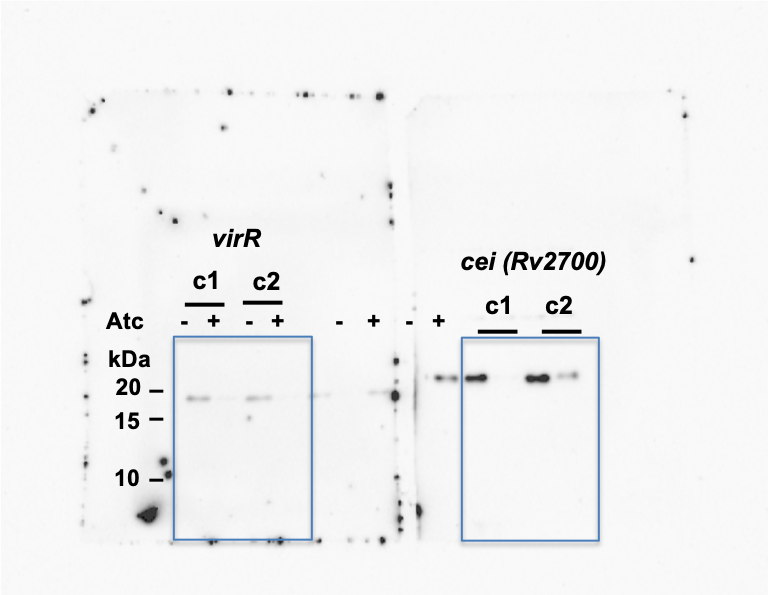

Supplement: Figure 2—source data 1. [file elife-94982-fig2-data1.zip › Figure_2_source_data_1/Figure_2D.png]

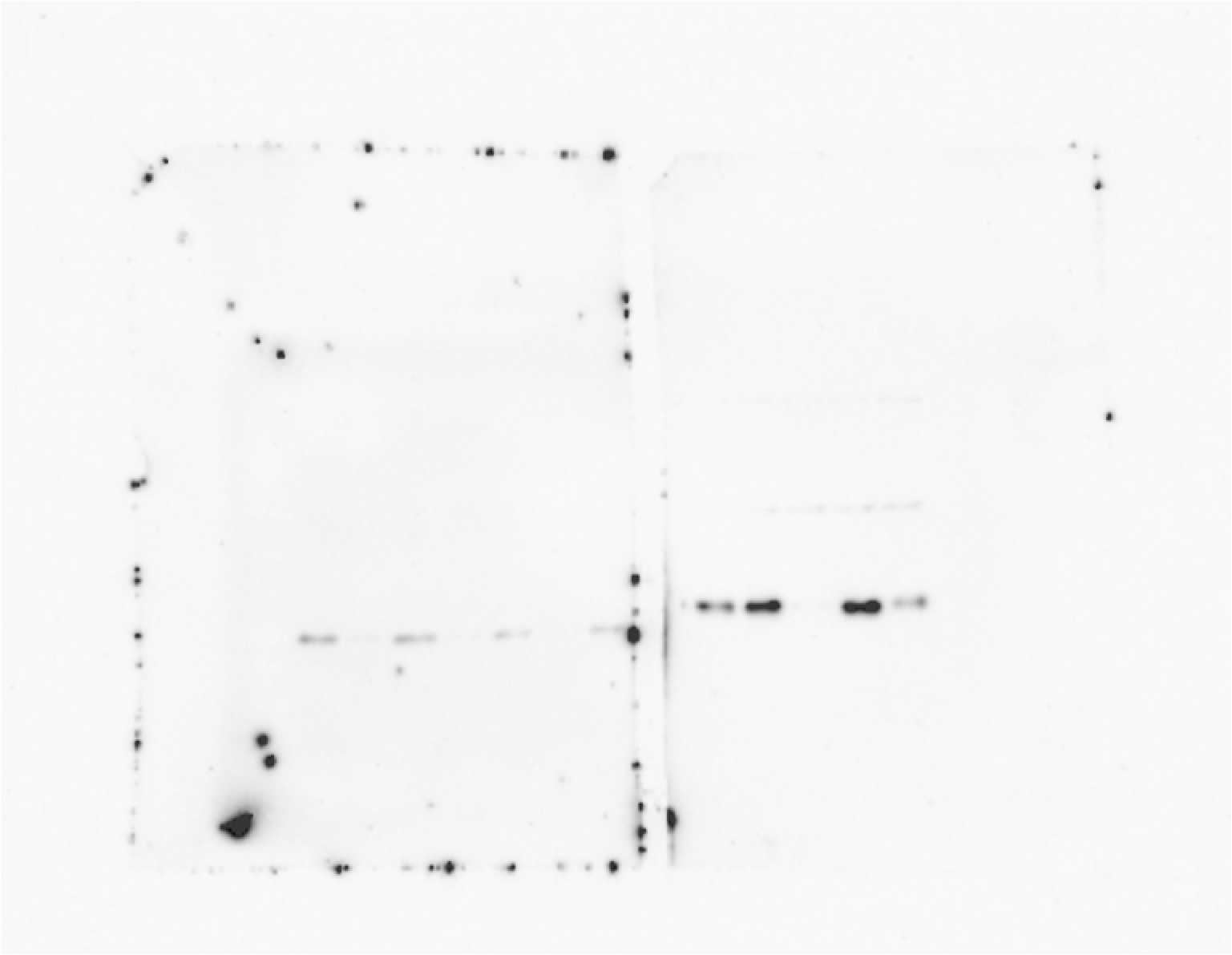

Supplement: Figure 2—source data 2. [file elife-94982-fig2-data2.zip › Figure_2_source_data_2/Figure_2D.tif]

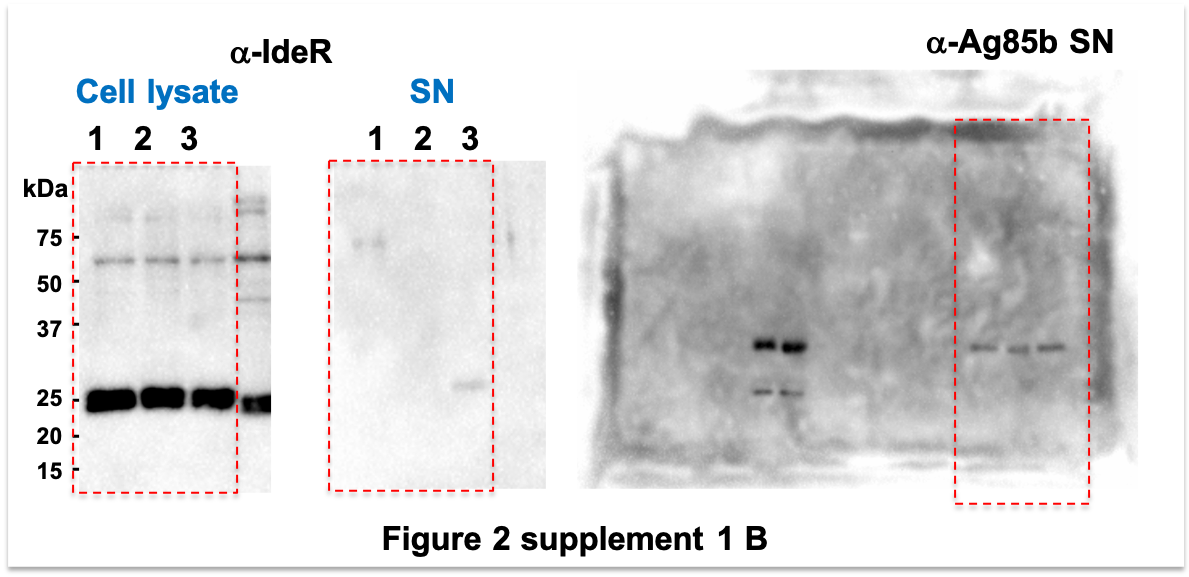

Supplement: Figure 2—figure supplement 1—source data 1. [file elife-94982-fig2-figsupp1-data1.zip › Figure_2_figure_supplement_1_source_data_1/Figure_2_figure_supplement_1B.png]

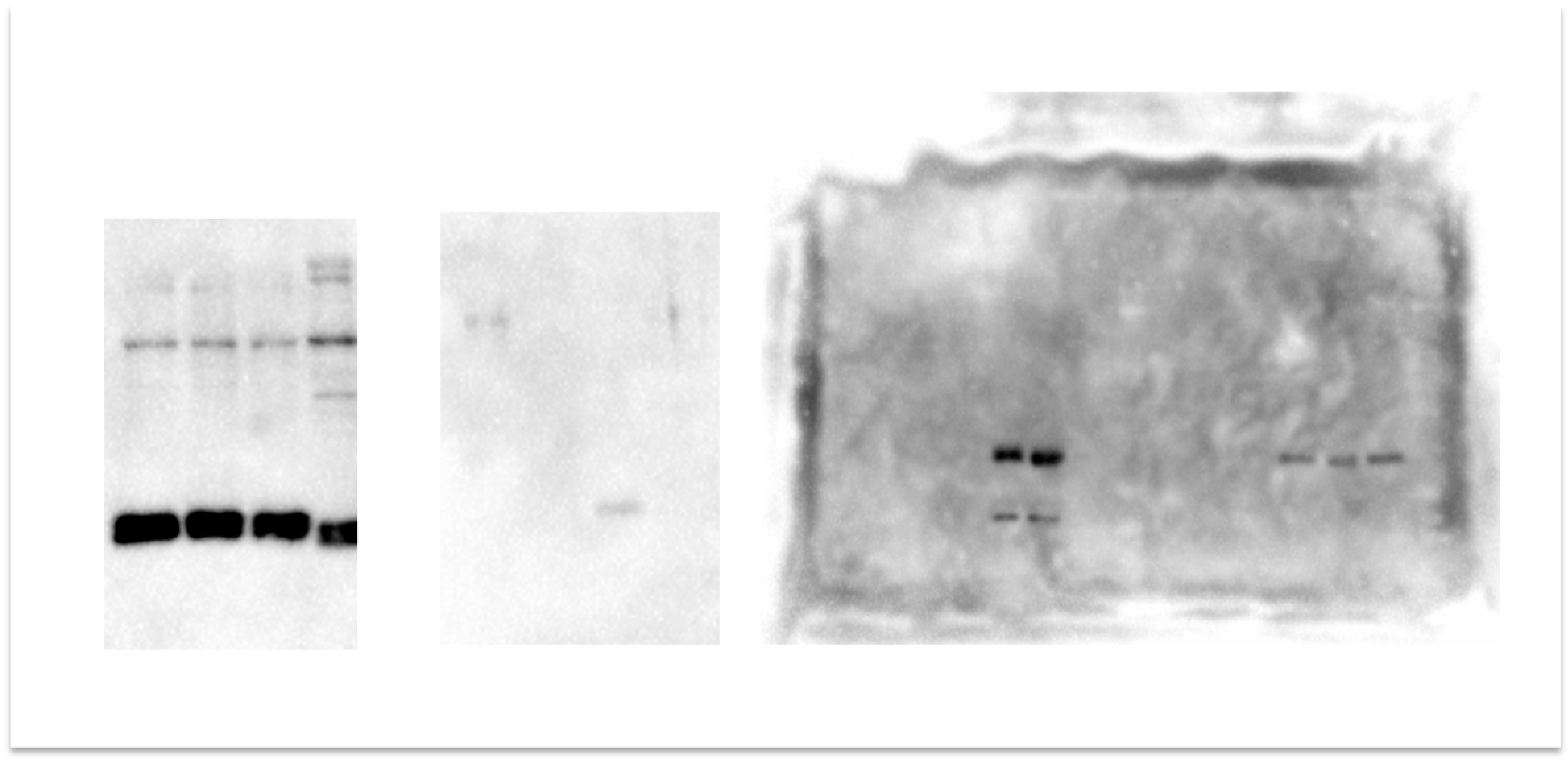

Supplement: Figure 2—figure supplement 1—source data 2. [file elife-94982-fig2-figsupp1-data2.zip › Figure_2_figure_supplement_1_source_data_2/Figure_2_figure_supplement_1B.tif]

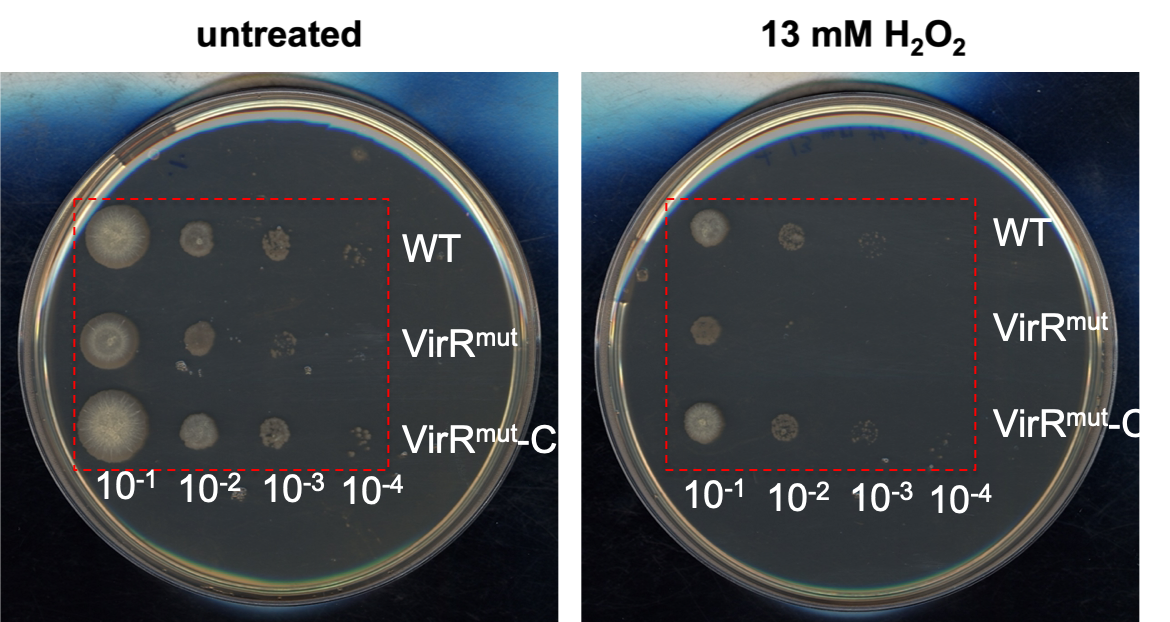

Supplement: Figure 4—figure supplement 1—source data 1. [file elife-94982-fig4-figsupp1-data1.zip › Figure_4_figure_supplement_1_source_data_1/Figure_4_figure_supplement_1.png]

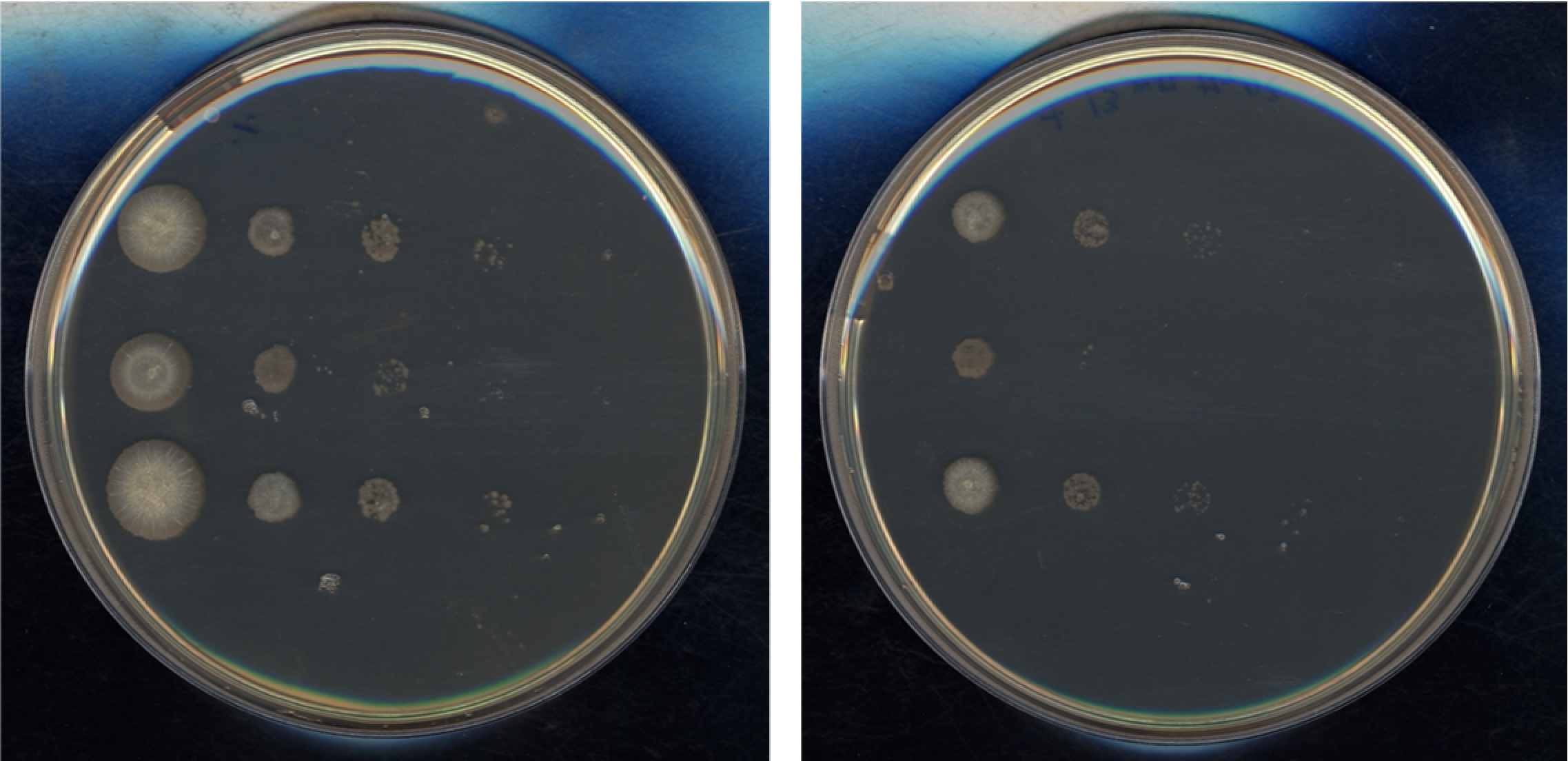

Supplement: Figure 4—figure supplement 1—source data 2. [file elife-94982-fig4-figsupp1-data2.zip › Figure_4_figure_supplement_1_source_data_2/Figure_4_figure_supplement_1.tif]

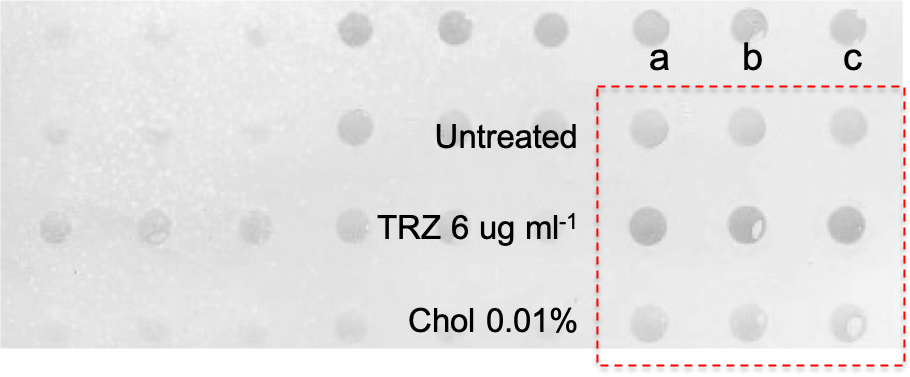

Supplement: Figure 5—source data 1. [file elife-94982-fig5-data1.zip › Figure_5_source_data_1/Figure_5B.png]

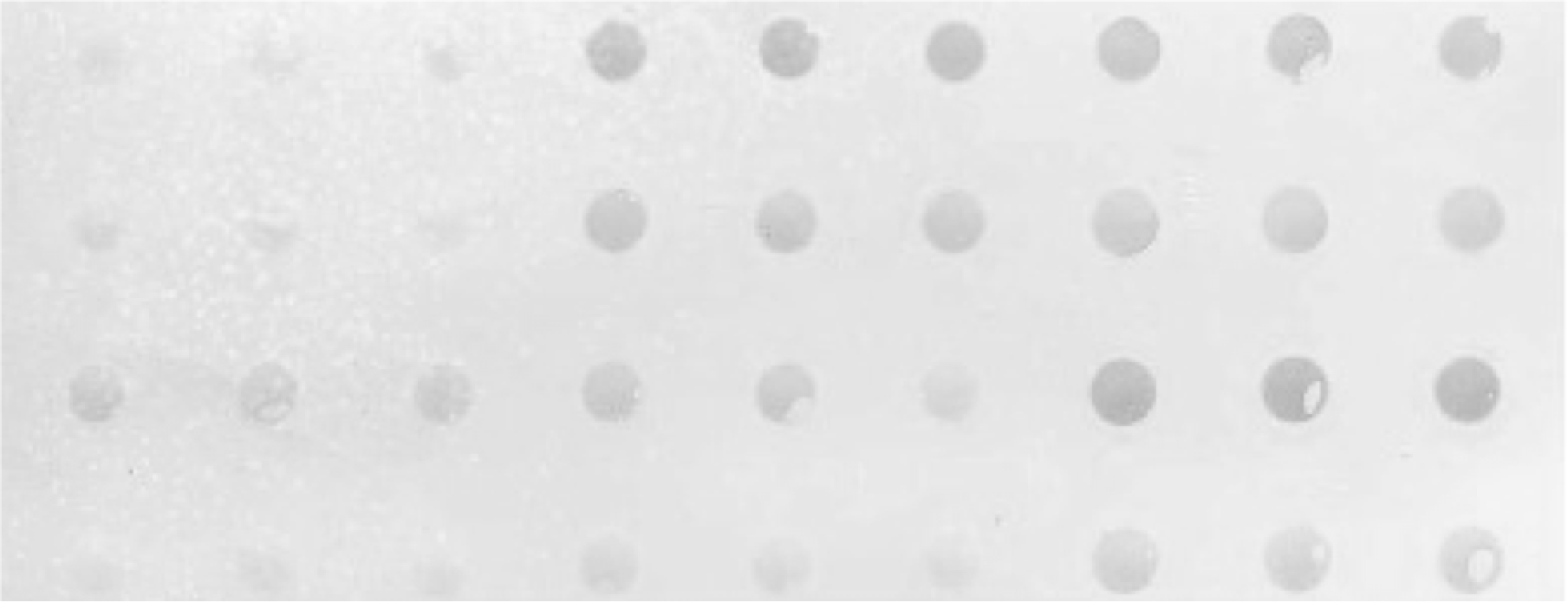

Supplement: Figure 5—source data 2. [file elife-94982-fig5-data2.zip › Figure_5_source_data_2/Figure_5B.tif]

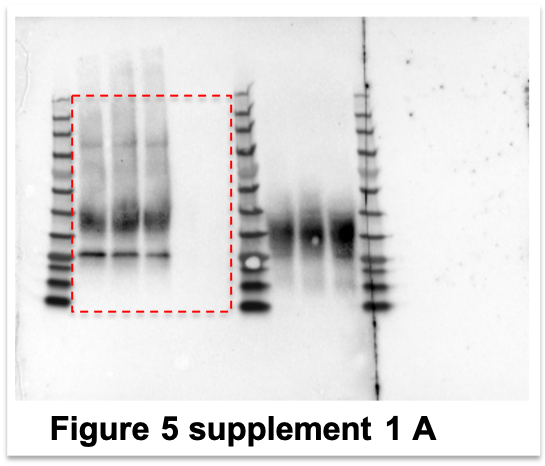

Supplement: Figure 5—figure supplement 1—source data 1. [file elife-94982-fig5-figsupp1-data1.zip › Figure_5_figure_supplement_1_source_data_1/Figure_5_figure_supplement_1A.png]

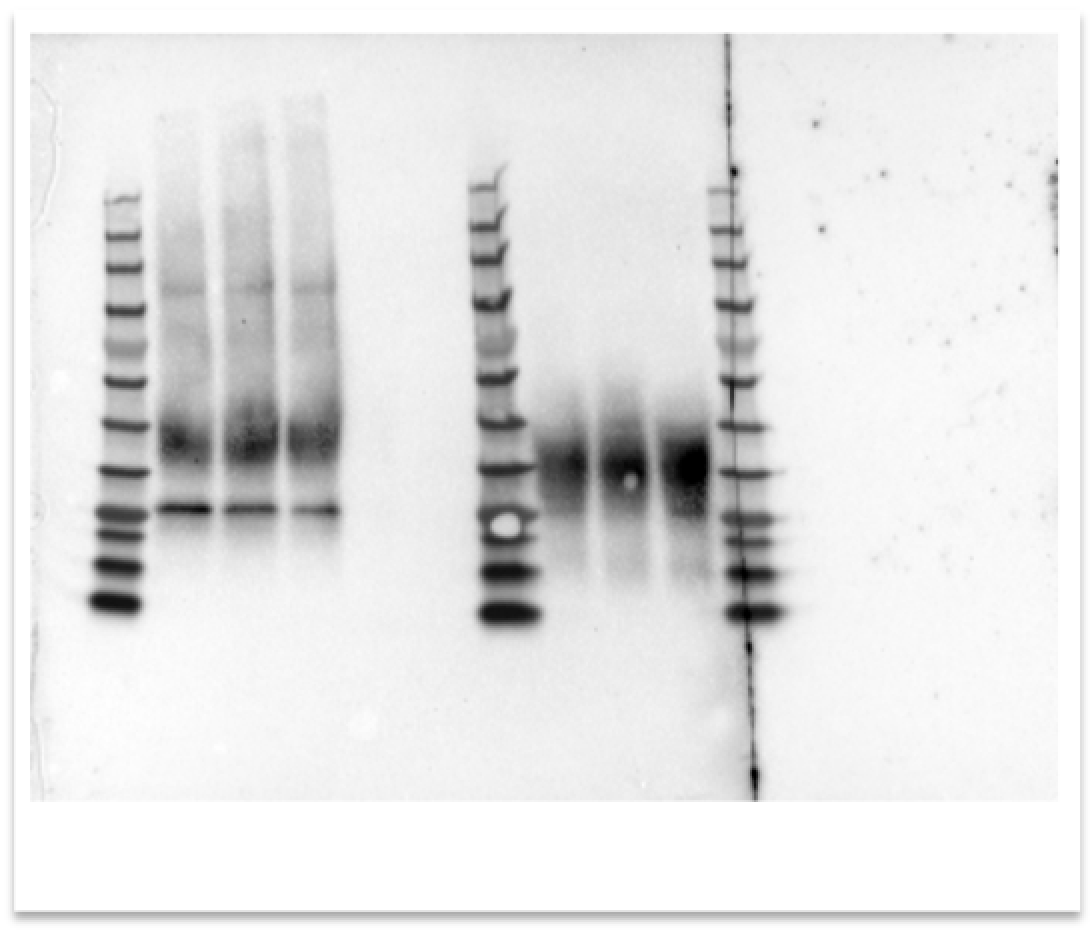

Supplement: Figure 5—figure supplement 1—source data 2. [file elife-94982-fig5-figsupp1-data2.zip › Figure_5_figure_supplement_1_source_data_2/Figure_5_figure_supplement_1A.tif]

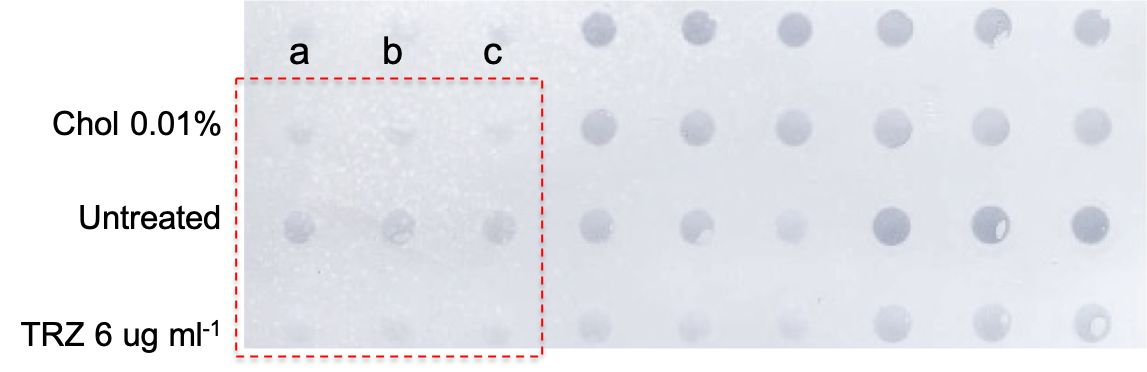

Supplement: Figure 5—figure supplement 3—source data 1. [file elife-94982-fig5-figsupp3-data1.zip › Figure_5_figure_supplement_3_source_data_1/Figure_5_figure_supplement_3B.png]

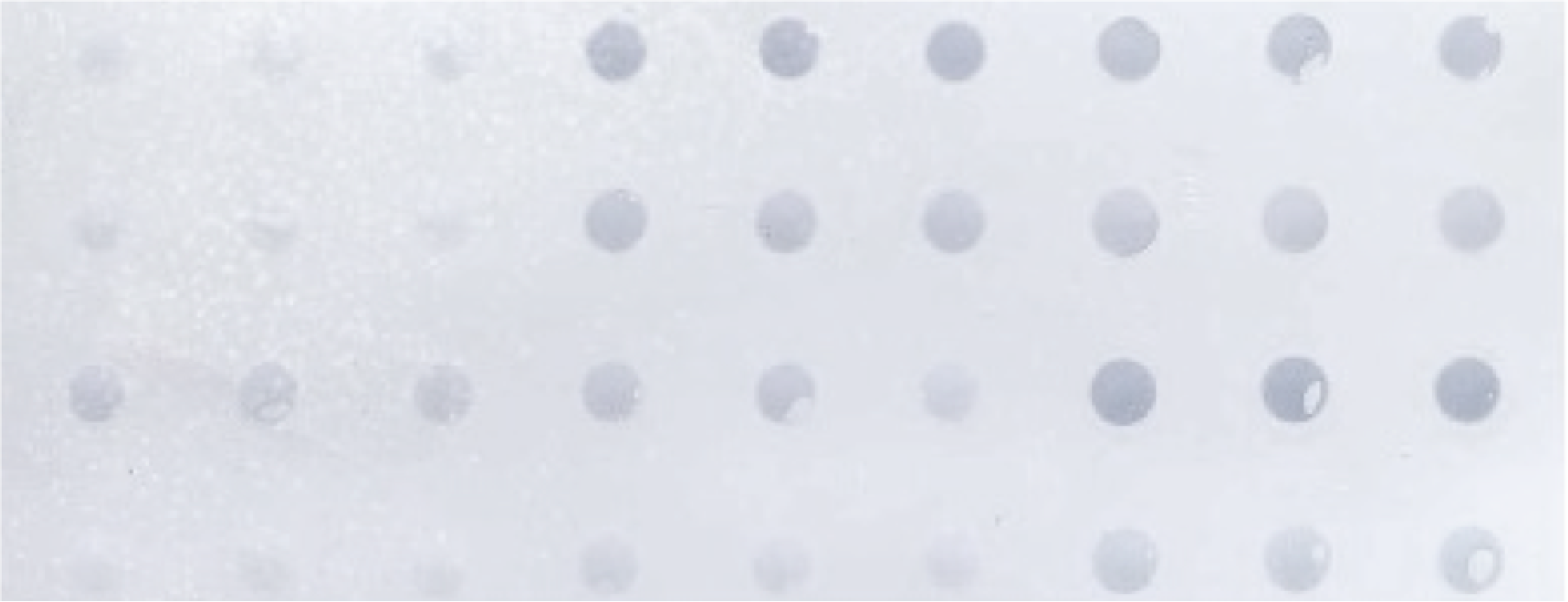

Supplement: Figure 5—figure supplement 3—source data 2. [file elife-94982-fig5-figsupp3-data2.zip › Figure_5_figure_supplement_3_source_data_2/Figure_5_figure_supplement_3B.tif]

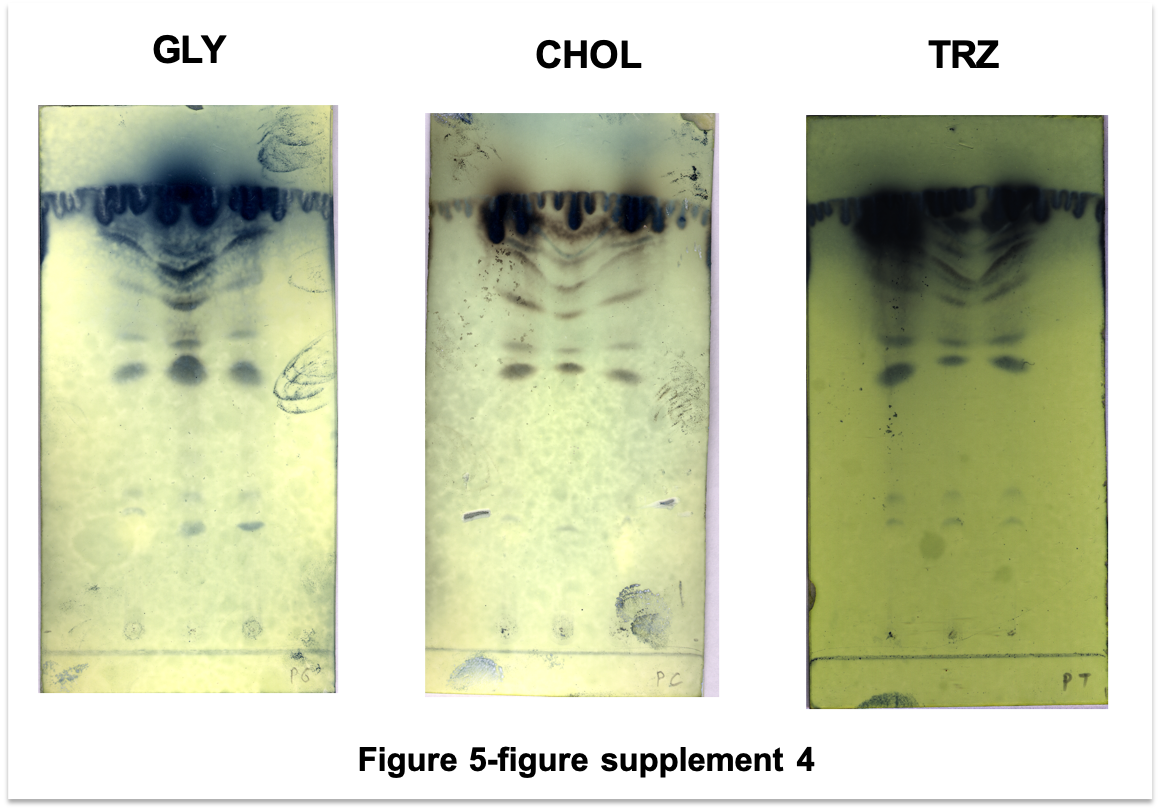

Supplement: Figure 5—figure supplement 4—source data 1. [file elife-94982-fig5-figsupp4-data1.zip › Figure_5_figure_supplement_4_source_data_1/Figure_5_figure_supplement_4.png]

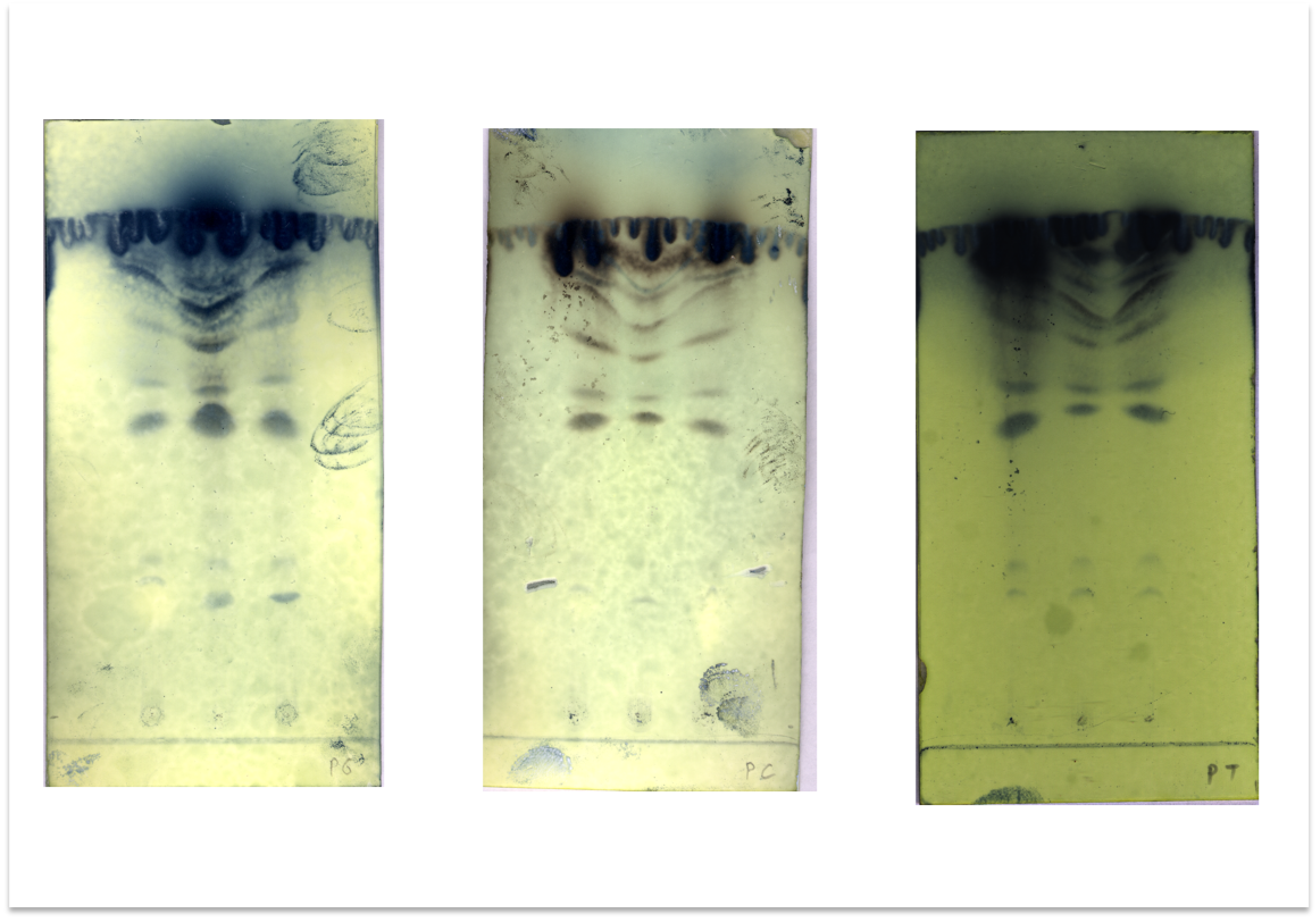

Supplement: Figure 5—figure supplement 4—source data 2. [file elife-94982-fig5-figsupp4-data2.zip › Figure_5_figure_supplement_4_source_data_2/Figure_5_figure_supplement_4.tif]

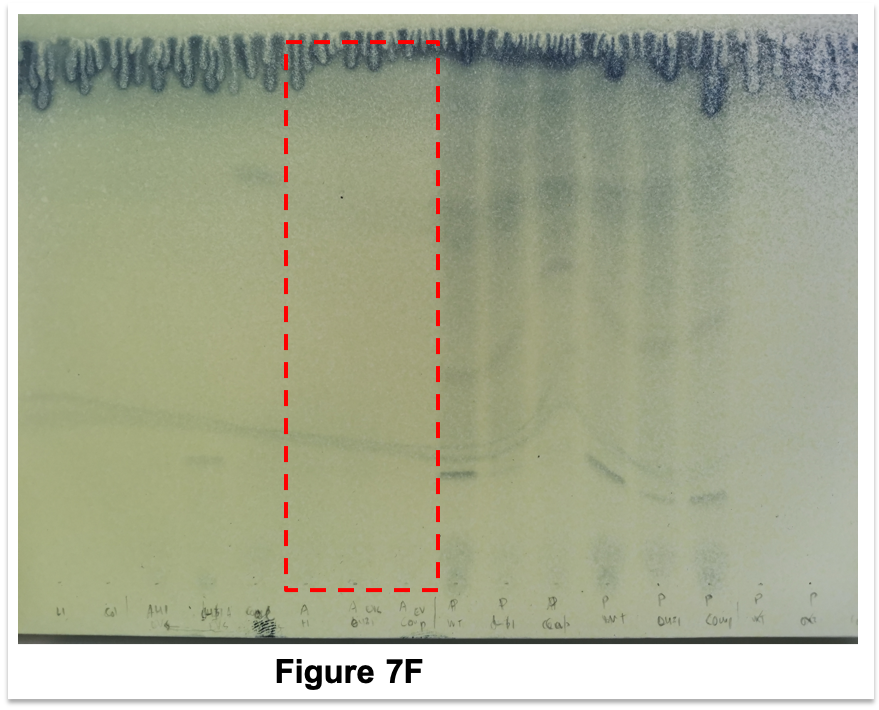

Supplement: Figure 7—source data 1. [file elife-94982-fig7-data1.zip › Figure_7_source_data_1/Figure_7F.png]

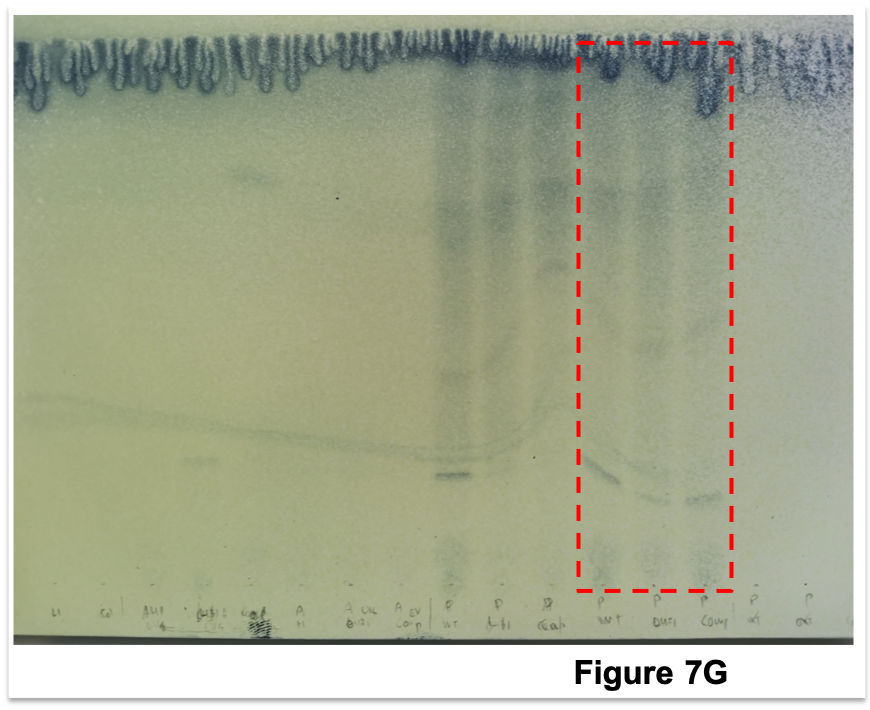

Supplement: Figure 7—source data 1. [file elife-94982-fig7-data1.zip › Figure_7_source_data_1/Figure_7G.png]

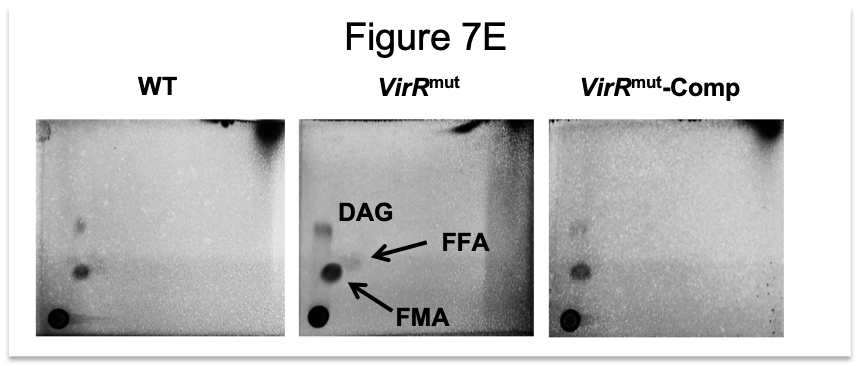

Supplement: Figure 7—source data 1. [file elife-94982-fig7-data1.zip › Figure_7_source_data_1/Figure_7E.png]

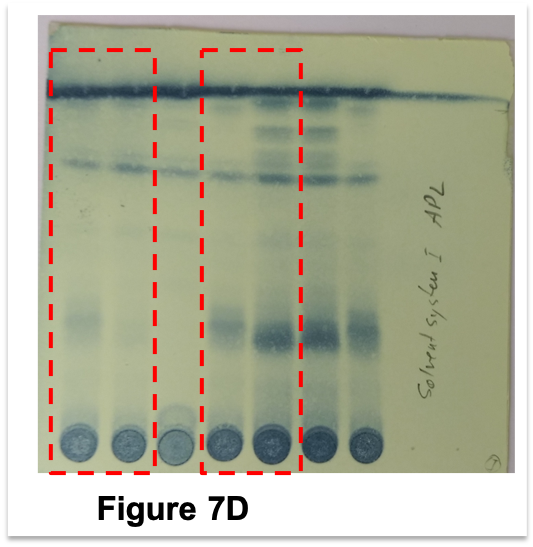

Supplement: Figure 7—source data 1. [file elife-94982-fig7-data1.zip › Figure_7_source_data_1/Figure_7D.png]

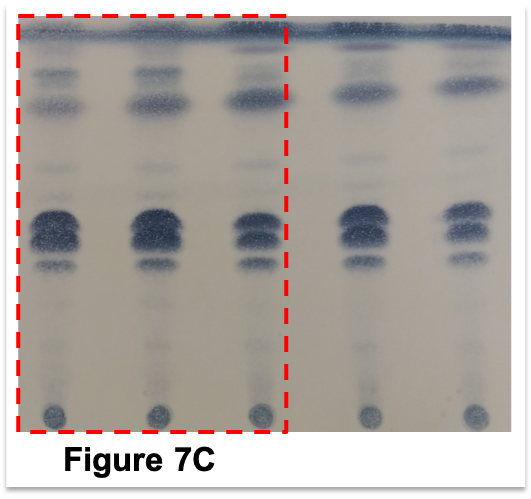

Supplement: Figure 7—source data 1. [file elife-94982-fig7-data1.zip › Figure_7_source_data_1/Figure_7C.png]

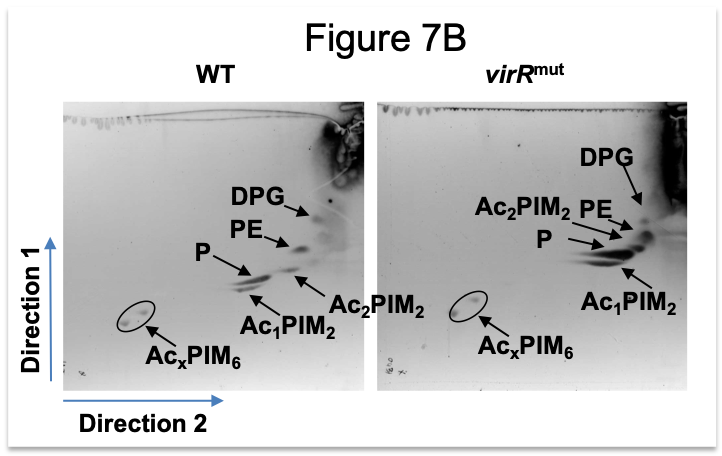

Supplement: Figure 7—source data 1. [file elife-94982-fig7-data1.zip › Figure_7_source_data_1/Figure_7B.png]

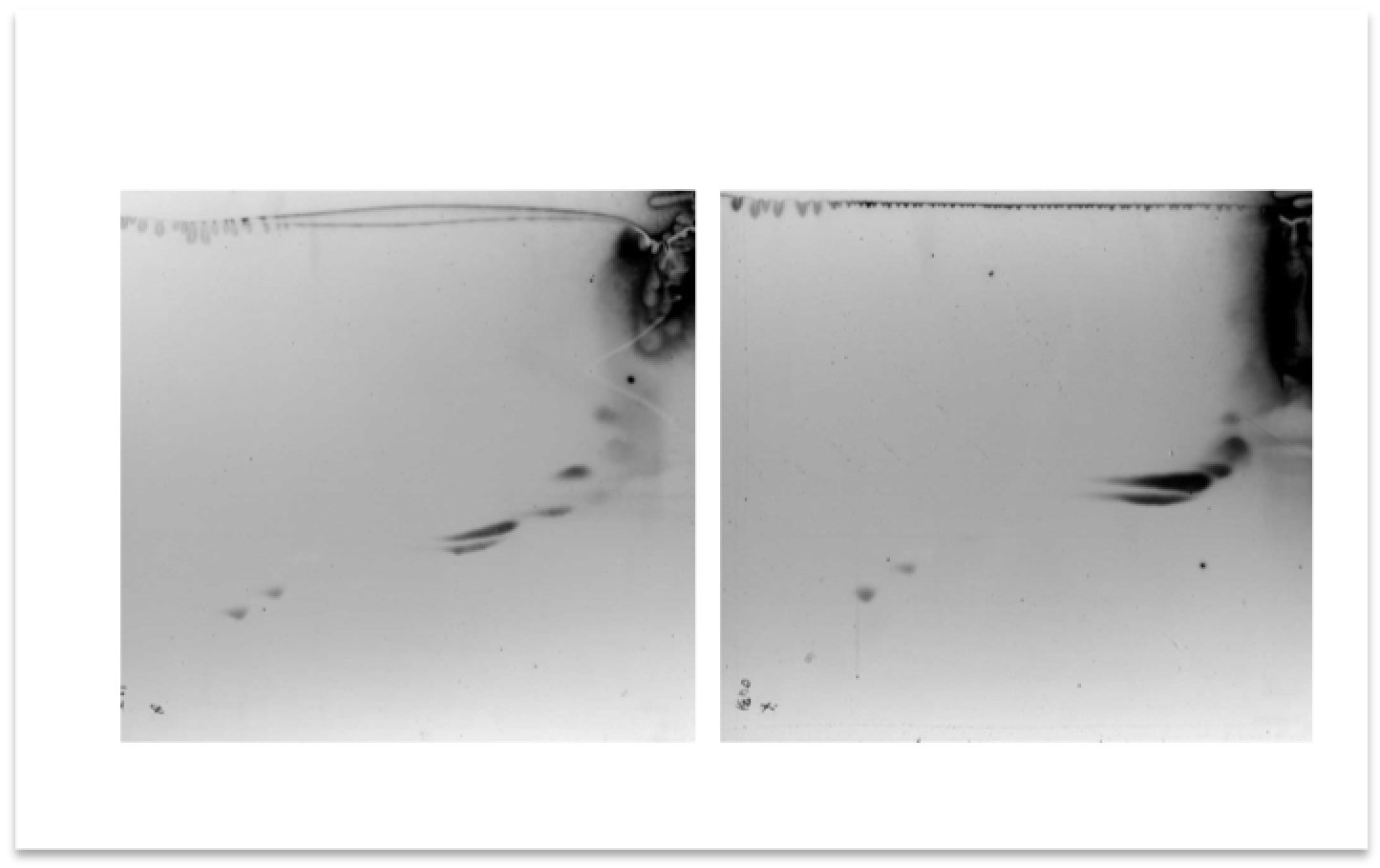

Supplement: Figure 7—source data 2. [file elife-94982-fig7-data2.zip › Figure_7_source_data_2/Figure_7B.tif]

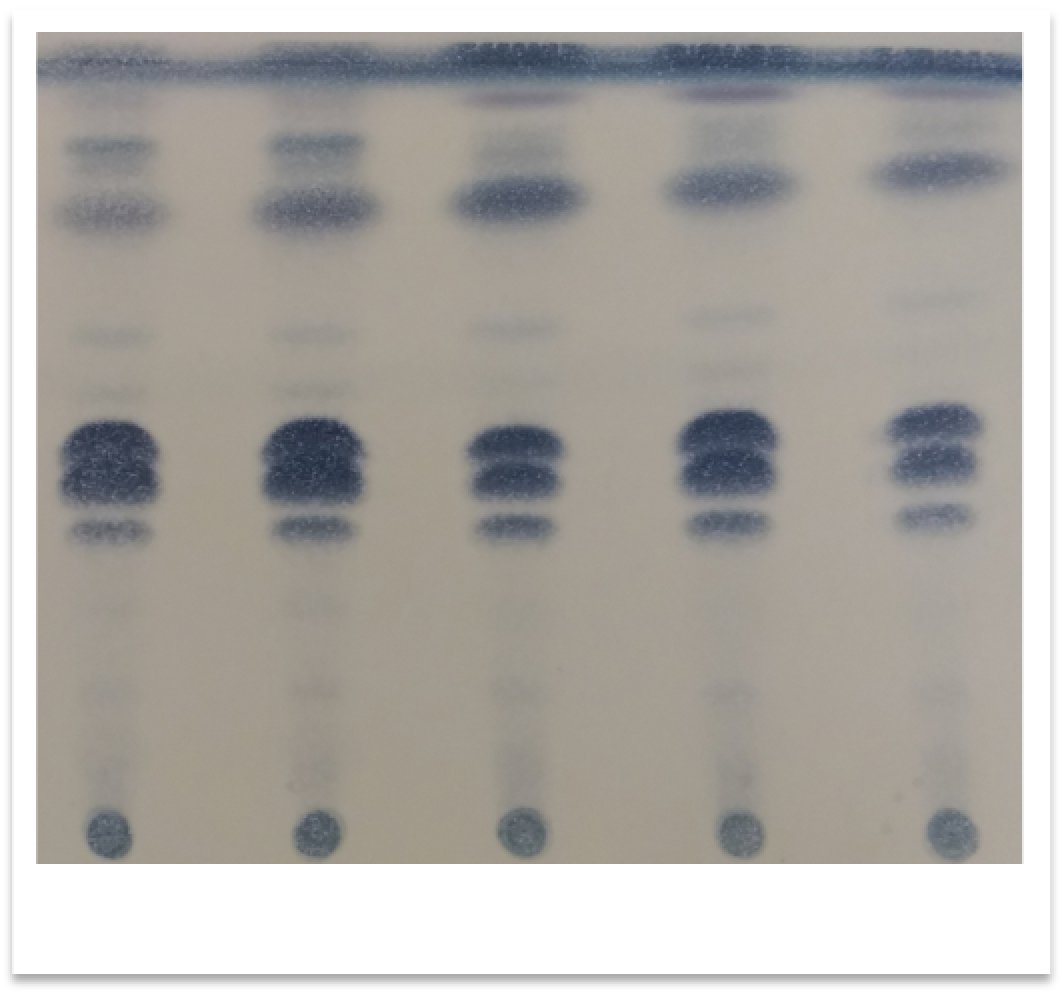

Supplement: Figure 7—source data 2. [file elife-94982-fig7-data2.zip › Figure_7_source_data_2/Figure_7C.tif]

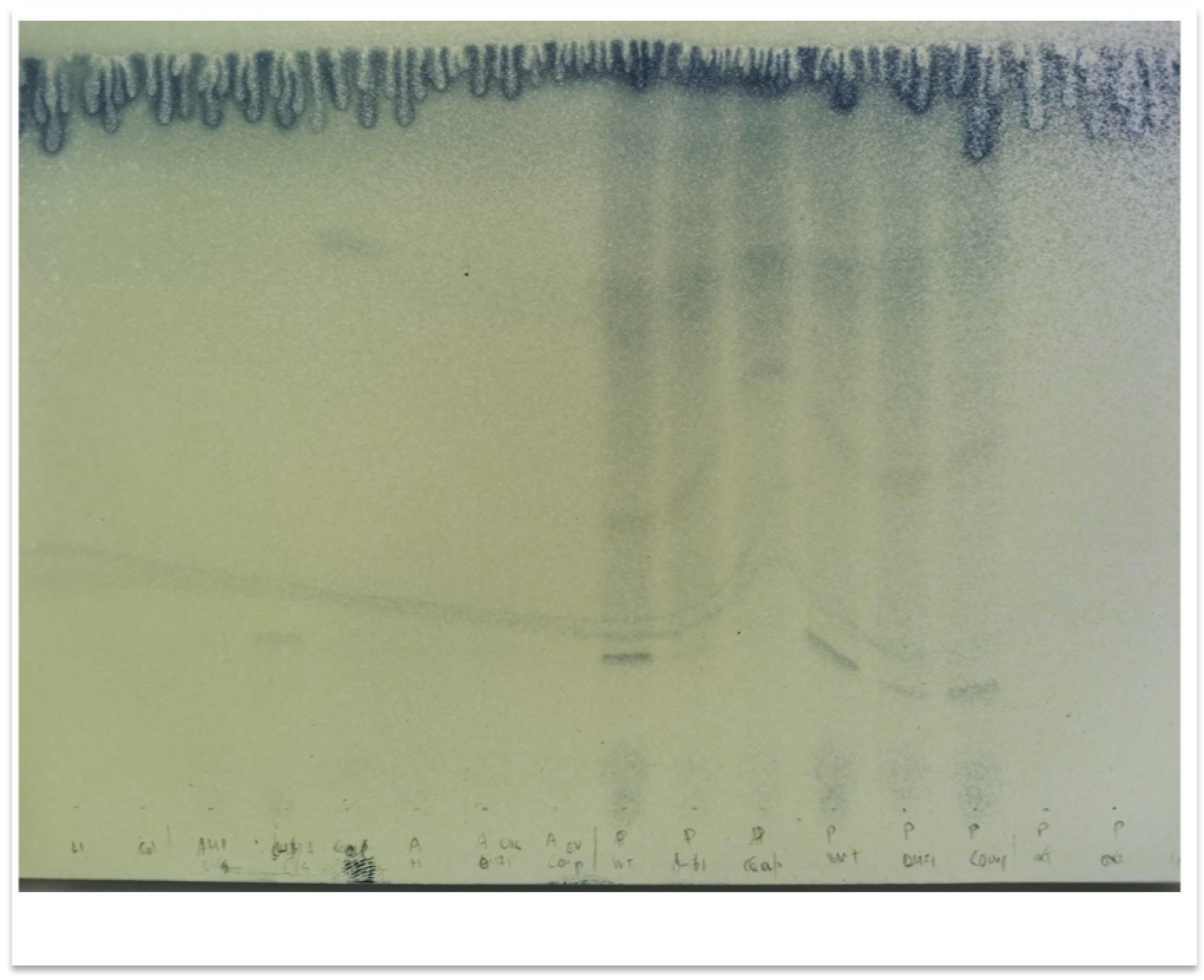

Supplement: Figure 7—source data 2. [file elife-94982-fig7-data2.zip › Figure_7_source_data_2/Figure_7G.tif]

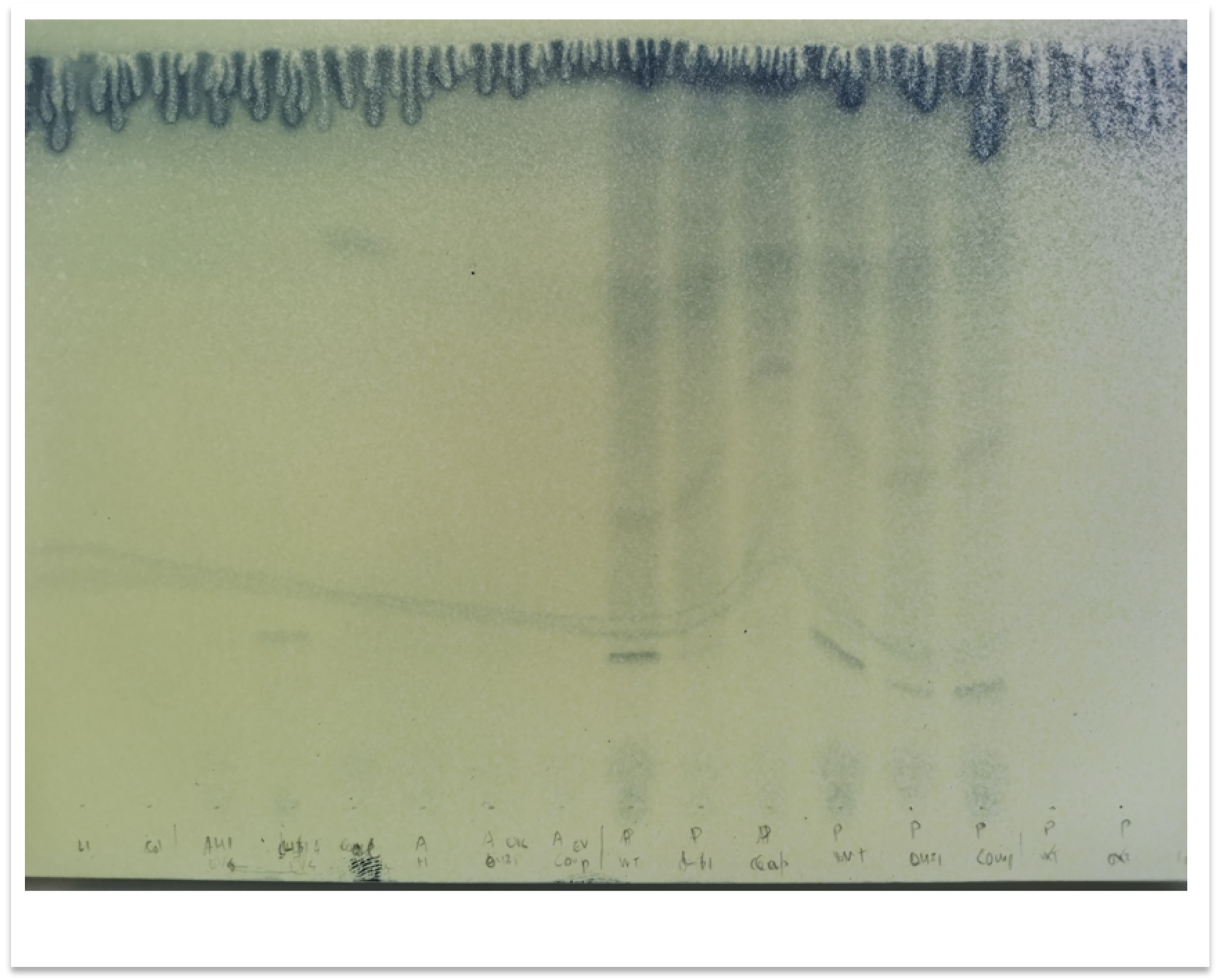

Supplement: Figure 7—source data 2. [file elife-94982-fig7-data2.zip › Figure_7_source_data_2/Figure_7F.tif]

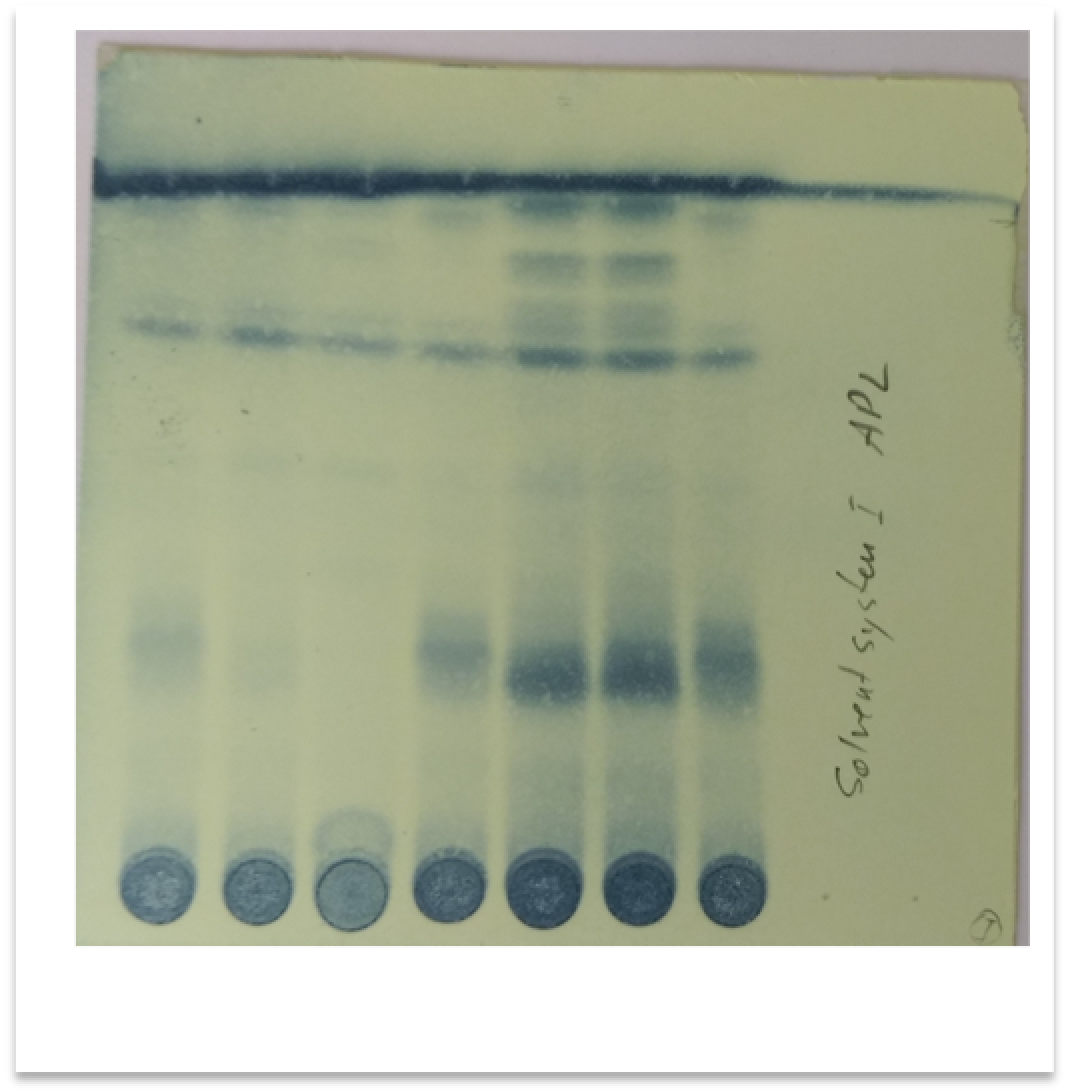

Supplement: Figure 7—source data 2. [file elife-94982-fig7-data2.zip › Figure_7_source_data_2/Figure_7D.tif]

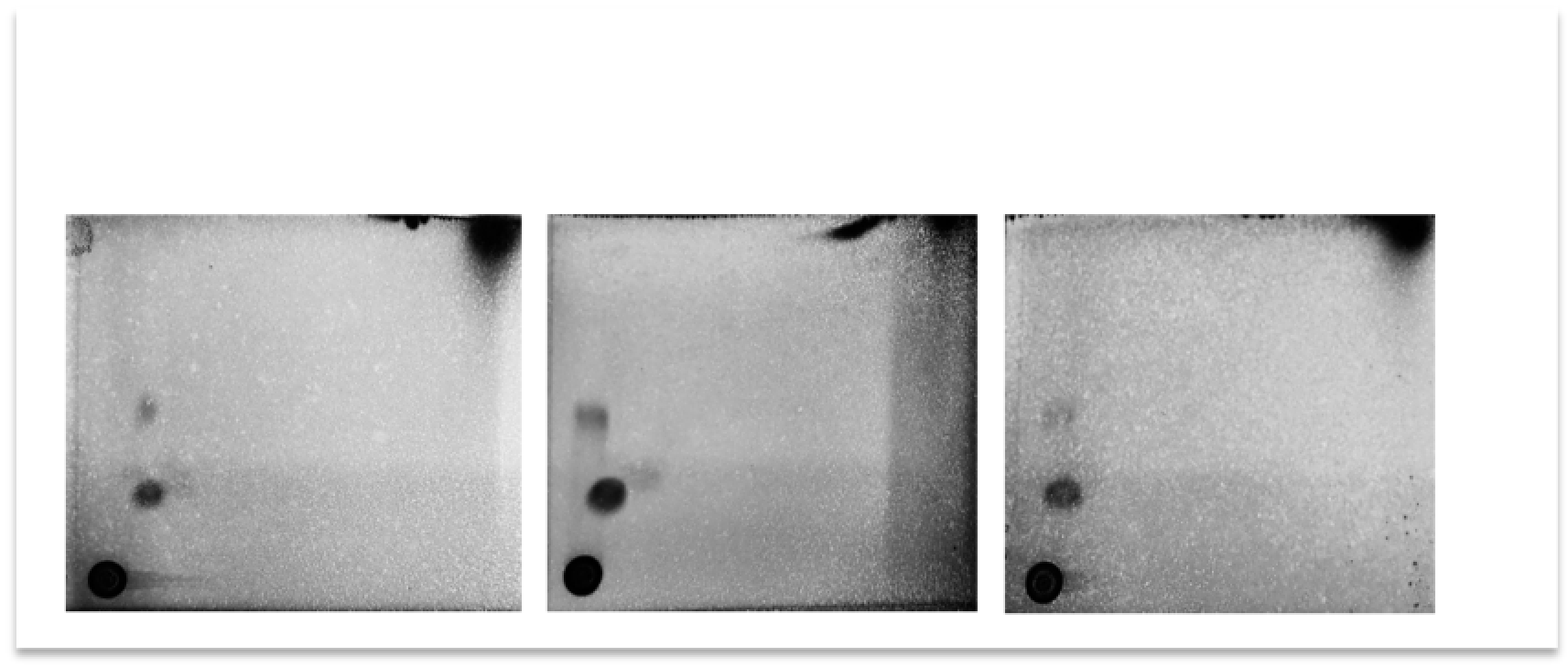

Supplement: Figure 7—source data 2. [file elife-94982-fig7-data2.zip › Figure_7_source_data_2/Figure_7E.tif]

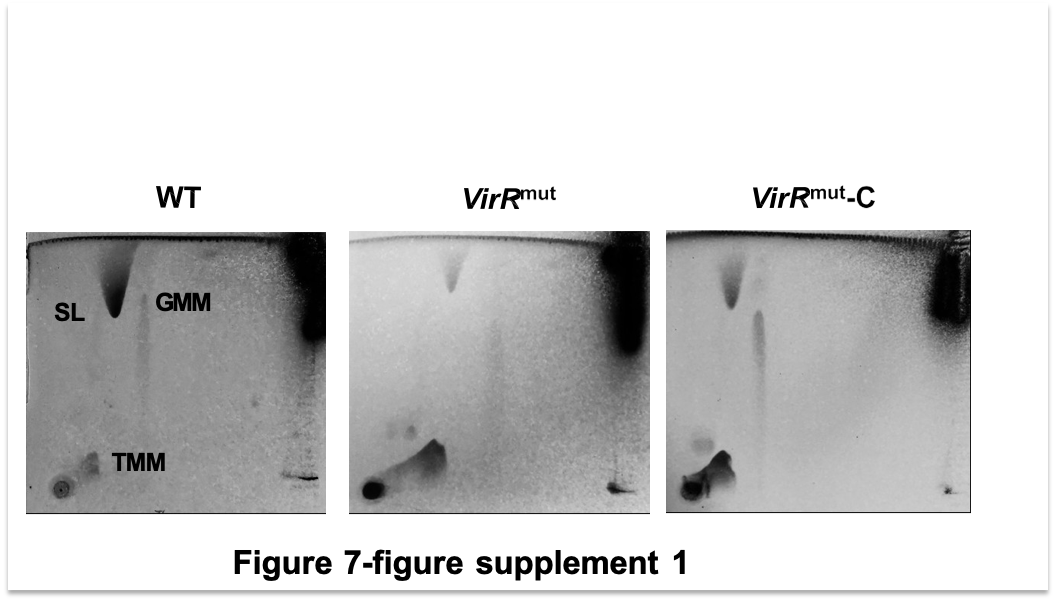

Supplement: Figure 7—figure supplement 1—source data 1. [file elife-94982-fig7-figsupp1-data1.zip › Figure_7_figure_supplement_1_source_data_1/Figure_7_figure_supplement_1.png]

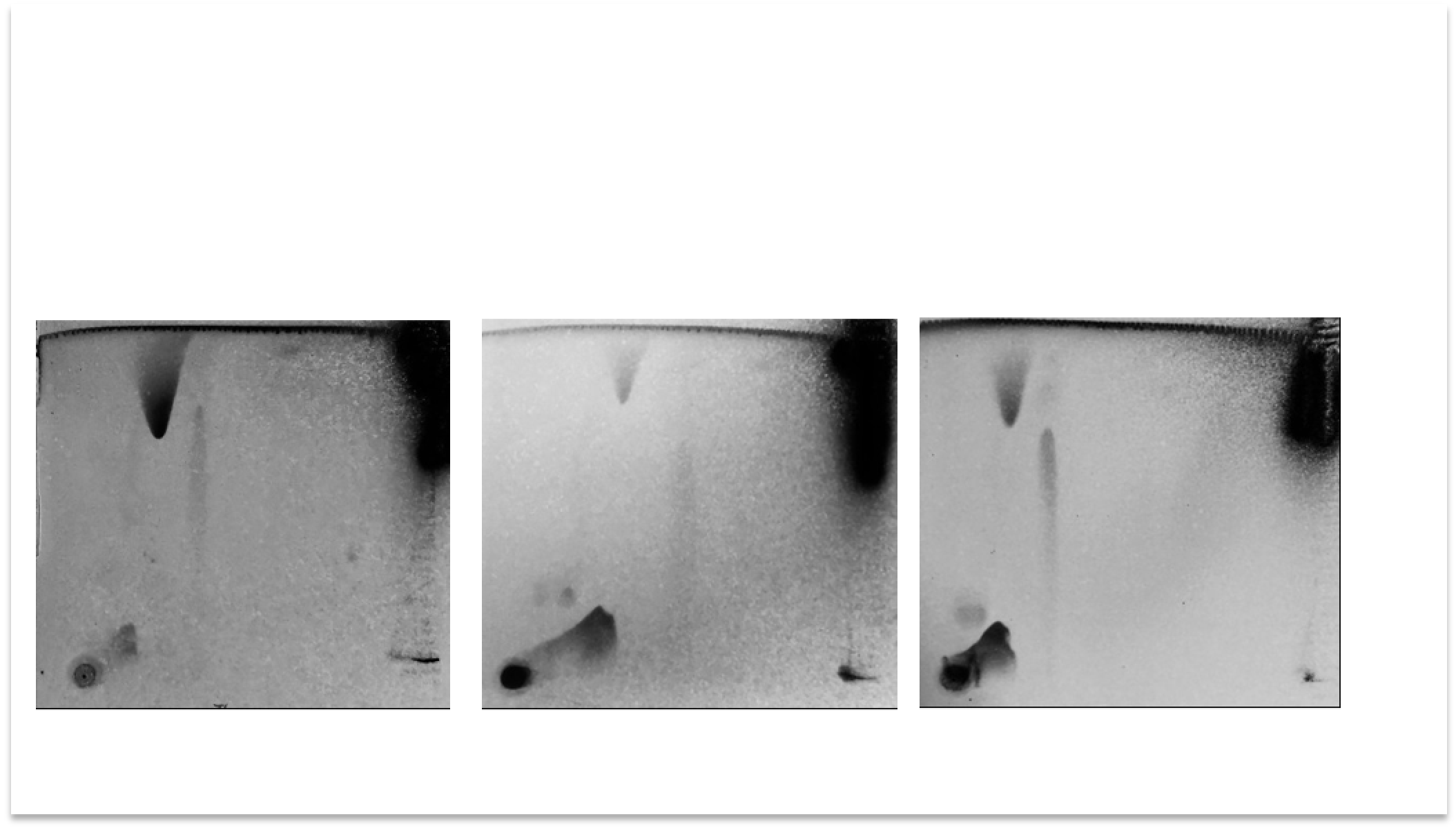

Supplement: Figure 7—figure supplement 1—source data 2. [file elife-94982-fig7-figsupp1-data2.zip › Figure_7_figure_supplement_1_source_data_2/Figure_7_figure_supplement_1.tif]

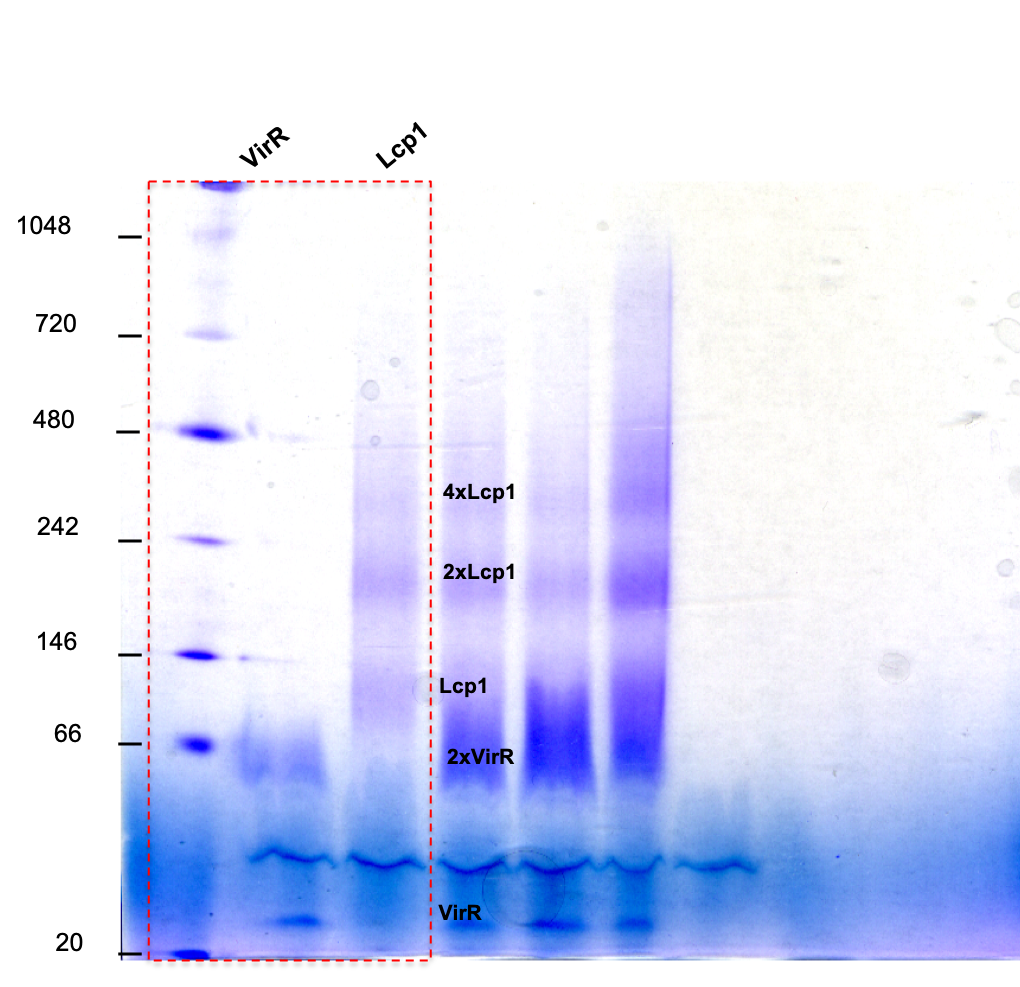

Supplement: Figure 9—source data 1. [file elife-94982-fig9-data1.zip › Figure_9_source_data_1/Figure_9A.png]

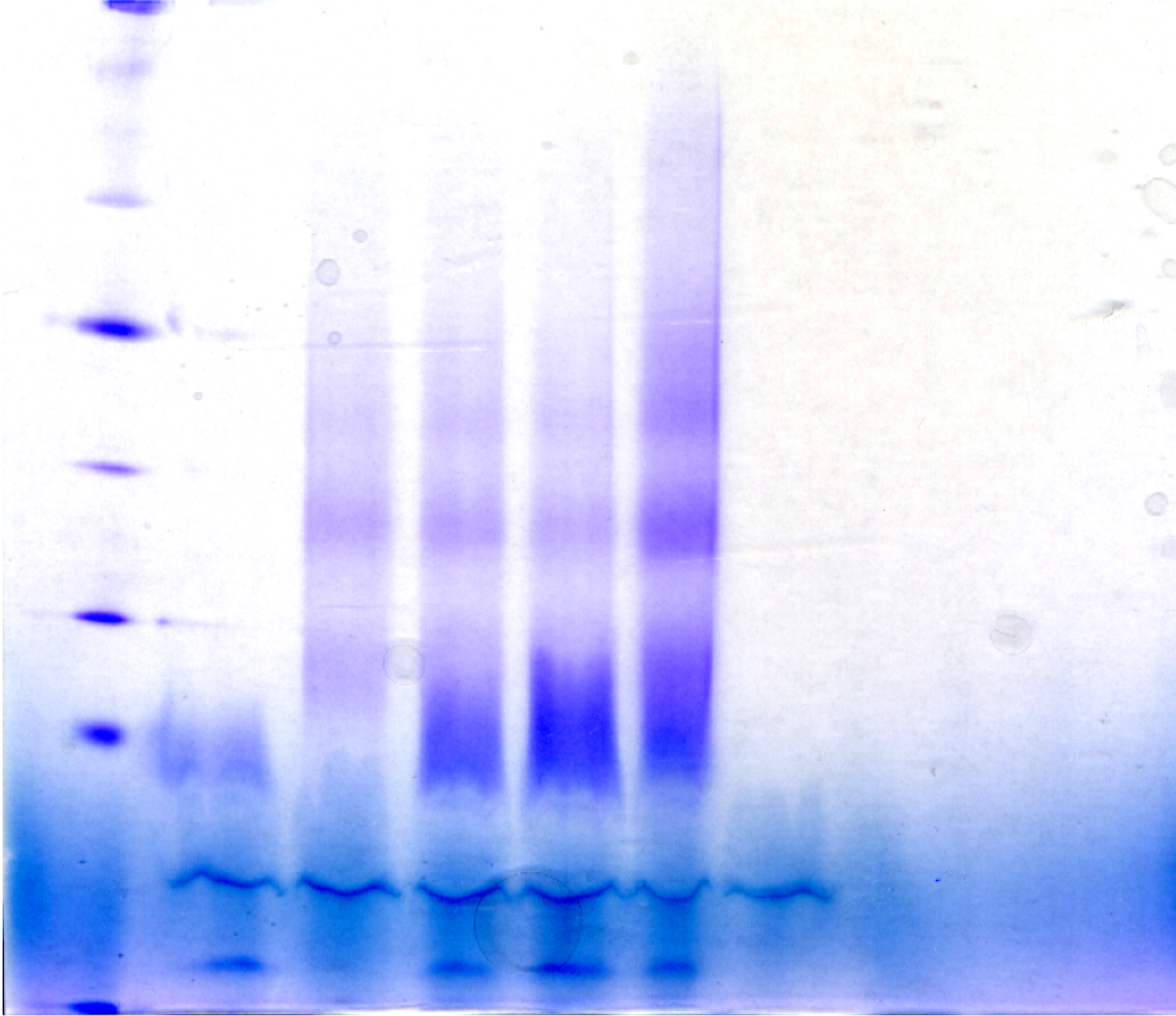

Supplement: Figure 9—source data 2. [file elife-94982-fig9-data2.zip › Figure_9_source_data_2/Figure_9A.tif]
