# Supplementary material for: Maintenance of cell wall remodeling and vesicle production are connected in Mycobacterium tuberculosis
Source: eLife. 2025 Feb 17;13:RP94982. doi: 10.7554/eLife.94982 (PMC11832169; doi:10.7554/eLife.94982)
Supplement: Supplementary file 32 [file elife-94982-supp6.zip › Supplement 6.odt]

Table supplement 1. Plasmids and primers used in this study.

| Construction / DNA fragment used | Template | Primers | Sequence 5’-3’ |
| --- | --- | --- | --- |
| pGMCS-P1-virR-Lk2-GFP11-ctpC(1-85)-Lk15-GFP(1-10) |
| P1 | pGMCS-TetR-P1- pacL1Flag- PpacL1-ctpCHis (Boudehen et al. 2022) | Inf-B2-P1-Fw | CAAAGTGGAGCGATACTCGACATCGGATCGTCGGCACCGTCAC |
| Inf-P1-Left | GAAATGATGTATGCCGTGCTGGTC |
| pGMCS backbone | pGMCS-PpacL-pacL1-Lk2-GFP11-ctpC(1-85)-Lk15-GFP(1-10) (Boudehen et al. 2022) | ctpC-B2-Left | GTCGAGTATCGCTCCACTTTG |
| Rv3269-P1-Fus-Fw | CAGCACGGCATACATCATTTCGGAAGAAAGGTACAGGCAATGG |
| pGMCS-P1-virR-Lk2-GFP11-ctpC(1-85)-Lk15-GFP(1-10) |
| virR | M. tuberculosis H37Rv chromosomal DNA | If-AmL1-VirR | AAGGTACAGGCAATGgtgctggttacagtgggctcg |
| If-Lk2-VirR-Rv | ACCGGATCCTTCCAGgccggtcaccacgacgatg |
| pGMCS backbone | pGMCS-P1-pacL1-Lk2-GFP11-ctpC(1-85)-Lk15-GFP(1-10) | Lk2-Right | CTGGAAGGATCCGGTGGCGG |
| pGMC 3269 Am Left | CATTGCCTGTACCTTTCTTCC |
| pGMCS-P1-virRsol-Lk2-GFP11-ctpC(1-85)-Lk15-GFP(1-10) |
| virRsol | M. tuberculosis H37Rv chromosomal DNA | If-AmL1-VirRsol | AAGGTACAGGCAATGctgggttcgtctccgaactccg |
| If-Lk2-VirR-Rv | ACCGGATCCTTCCAGgccggtcaccacgacgatg |
| pGMCS backbone | pGMCS-P1-pacL1-Lk2-GFP11-ctpC(1-85)-Lk15-GFP(1-10) | Lk2-Right | CTGGAAGGATCCGGTGGCGG |
| pGMC 3269 Am Left | CATTGCCTGTACCTTTCTTCC |
| pGMCS-P1-virR-Lk2-GFP11-virR-Lk15-GFP(1-10) |
| virR | M. tuberculosis H37Rv chromosomal DNA | If-AmMBD-VirR | GAGCGCCTCGCCATGgtgctggttacagtgggctcg |
| If-L15-VirR-Rv | AGACAGCCCTGGACCgccggtcaccacgacgatg |
| pGMCS backbone | pGMCS-P1-virR-Lk2-GFP11-ctpC(1-85)-Lk15-GFP(1-10) | Flex15-Right | GGTCCAGGGCTGTCTGGCC |
| ctpC-Am-left2 | CATGGCGAGGCGCTCAAG |
| pGMCS-P1-virRsol-Lk2-GFP11-virRsol-Lk15-GFP(1-10) |
| virRsol | M. tuberculosis H37Rv chromosomal DNA | If-AmMBD-VirRsol | GAGCGCCTCGCCATGctgggttcgtctccgaactccg |
| If-L15-VirR-Rv | AGACAGCCCTGGACCgccggtcaccacgacgatg |
| pGMCS backbone | pGMCS-P1-virRsol-Lk2-GFP11-ctpC(1-85)-Lk15-GFP(1-10) | Flex15-Right | GGTCCAGGGCTGTCTGGCC |
| ctpC-Am-left2 | CATGGCGAGGCGCTCAAG |
| pGMCS-P1-virRsol-Lk2-GFP11-rv0822c(LytR_C)-Lk15-GFP(1-10) |
| Rv0822c(LytR_C) | M. tuberculosis H37Rv chromosomal DNA | If-AmMBD-0822cD | GAGCGCCTCGCCATGgagattcagcaccagcaggttacgacg |
| If-L15-0822cD-Rv | AGACAGCCCTGGACCgcggcttatctgcacgctgacg |
| pGMCS backbone | pGMCS-P1-virRsol-Lk2-GFP11-ctpC(1-85)-Lk15-GFP(1-10) | Flex15-Right | GGTCCAGGGCTGTCTGGCC |
| ctpC-Am-left2 | CATGGCGAGGCGCTCAAG |
| pGMCS-P1-virRsol-Lk2-GFP11-rv3267(LytR_C)-Lk15-GFP(1-10) |
| Rv3267(LytR_C) | M. tuberculosis H37Rv chromosomal DNA | If-AmMBD-3267D | GAGCGCCTCGCCATGcccgccaagaccacggcc |
| If-L15-3267D-Rv | AGACAGCCCTGGACCcagcccggagcccggac |
| pGMCS backbone | pGMCS-P1-virRsol-Lk2-GFP11-ctpC(1-85)-Lk15-GFP(1-10) | Flex15-Right | GGTCCAGGGCTGTCTGGCC |
| ctpC-Am-left2 | CATGGCGAGGCGCTCAAG |
| pGMCS-P1-virRsol-Lk2-GFP11-rv3484(LytR_C)-Lk15-GFP(1-10) |
| Rv3484(LytR_C) | M. tuberculosis H37Rv chromosomal DNA | If-AmMBD-3484D | GAGCGCCTCGCCATGgcggccgccaaacctaaccc |
| If-L15-3484D-Rv | AGACAGCCCTGGACCggtatccaccgtcacacggatg |
| pGMCS backbone | pGMCS-P1-virRsol-Lk2-GFP11-ctpC(1-85)-Lk15-GFP(1-10) | Flex15-Right | GGTCCAGGGCTGTCTGGCC |
| ctpC-Am-left2 | CATGGCGAGGCGCTCAAG |
| pGMCS-P1-virRsol-Lk2-GFP11-rv0822c(Cterm)-Lk15-GFP(1-10) |
| Rv0822c(Cterm) | M. tuberculosis H37Rv chromosomal DNA | If-AmMBD-0822cD | GAGCGCCTCGCCATGgagattcagcaccagcaggttacgacg |
| If-L15-0822c-Rv2 | AGACAGCCCTGGACCctcgcaggtggtgtcggcg |
| pGMCS backbone | pGMCS-P1-virRsol-Lk2-GFP11-ctpC(1-85)-Lk15-GFP(1-10) | Flex15-Right | GGTCCAGGGCTGTCTGGCC |
| ctpC-Am-left2 | CATGGCGAGGCGCTCAAG |
| pGMCS-P1-virRsol-Lk2-GFP11-rv3267(Cterm)-Lk15-GFP(1-10) |
| Rv0822c(Cterm) | M. tuberculosis H37Rv chromosomal DNA | If-AmMBD-3267D | GAGCGCCTCGCCATGgagattcagcaccagcaggttacgacg |
| If-L15-3267-Rv2 | AGACAGCCCTGGACCgttgatgcactccggcgcgtc |
| pGMCS backbone | pGMCS-P1-virRsol-Lk2-GFP11-ctpC(1-85)-Lk15-GFP(1-10) | Flex15-Right | GGTCCAGGGCTGTCTGGCC |
| ctpC-Am-left2 | CATGGCGAGGCGCTCAAG |
| pGMCS-P1-virRsol-Lk2-GFP11-rv3484(Cterm)-Lk15-GFP(1-10) |
| Rv0822c(Cterm) | M. tuberculosis H37Rv chromosomal DNA | If-AmMBD-3267D | GAGCGCCTCGCCATGgagattcagcaccagcaggttacgacg |
| If-L15-3484-Rv2 | AGACAGCCCTGGACCgttcacgcagggcacgccg |
| pGMCS backbone | pGMCS-P1-virRsol-Lk2-GFP11-ctpC(1-85)-Lk15-GFP(1-10) | Flex15-Right | GGTCCAGGGCTGTCTGGCC |
| ctpC-Am-left2 | CATGGCGAGGCGCTCAAG |
| pGMCS-P1-virRsol-Lk2-GFP11-rv0822c-Lk15-GFP(1-10) |
| Rv0822c | M. tuberculosis H37Rv chromosomal DNA | If-AmMBD-0822c | GAGCGCCTCGCCATGagtgacggcgagagcg |
| If-L15-0822c-Rv | AGACAGCCCTGGACCctactcgcaggtggtgtcgg |
| pGMCS backbone | pGMCS-P1-virRsol-Lk2-GFP11-ctpC(1-85)-Lk15-GFP(1-10) | Flex15-Right | GGTCCAGGGCTGTCTGGCC |
| ctpC-Am-left2 | CATGGCGAGGCGCTCAAG |
| pGMCS-P1-virRsol-Lk2-GFP11-rv3267-Lk15-GFP(1-10) |
| Rv3267(Cterm) | M. tuberculosis H37Rv chromosomal DNA | If-AmMBD-3267 | GAGCGCCTCGCCATGatgtctgcgcaacgtgtggttc |
| If-L15-3267-Rv | AGACAGCCCTGGACCtcagttgatgcactccggcgc |
| pGMCS backbone | pGMCS-P1-virRsol-Lk2-GFP11-ctpC(1-85)-Lk15-GFP(1-10) | Flex15-Right | GGTCCAGGGCTGTCTGGCC |
| ctpC-Am-left2 | CATGGCGAGGCGCTCAAG |
| pGMCS-P1-virRsol-Lk2-GFP11-rv3484-Lk15-GFP(1-10) |
| Rv3484(Cterm) | M. tuberculosis H37Rv chromosomal DNA | If-AmMBD-3484 | GAGCGCCTCGCCATGgcgcgttctgagggc |
| If-L15-3484-Rv | AGACAGCCCTGGACCctagttcacgcagggcacgc |
| pGMCS backbone | pGMCS-P1-virRsol-Lk2-GFP11-ctpC(1-85)-Lk15-GFP(1-10) | Flex15-Right | GGTCCAGGGCTGTCTGGCC |
| ctpC-Am-left2 | CATGGCGAGGCGCTCAAG |
| CRISPRi mutants |
| virR (158.1) c2 | Rv0431 | Rv0431crispr 1 F | GGGAGAGCAGCAGGAAGACGACGC |
| Rv0431crispr 1 R | AAACGCGTCGTCTTCCTGCTGCTC |
| virR (42.2) c1 | Rv0431 | Rv0431crispr 2 F | GGGAACGAACCCAGTGCCTGCCAGA |
| Rv0431crispr 2 R | AAACtctggcaggcactgggttcgt |
| cei (145.2) c1 | Rv2700 | Rv2700crispr 1 F | GGGAATCACGCAAGTCACCACCAC |
| Rv2700crispr 1 R | AAACGTGGTGGTGACTTGCGTGAT |
| cei (47.3) c2 | Rv2700 | Rv2700crispr 2 F | gggagtcggtgccgagcgcaaggtcg |
| Rv2700crispr 2 R | aaacgaccttgcgctcggcaccgac |
| VirR-Venus primers |
| VirR-F | TCACTGGGGTGCTGGTTACAGTGGGCTC |
| VirR-R | ATAGGATCGCCGGTCACCACGACGAT |
| pMV-F | TGACCGGCGATCCTATGGTGAGCAAGGGC |
| pMV-R | ACCAGCACCCCAGTGAAAAGTTCTTCTCCTTTACTC |
