# Supplementary material for: Maintenance of cell wall remodeling and vesicle production are connected in Mycobacterium tuberculosis
Source: eLife. 2025 Feb 17;13:RP94982. doi: 10.7554/eLife.94982 (PMC11832169; doi:10.7554/eLife.94982)
Supplement: Supplementary file 33 [file elife-94982-supp7.zip › Supplement 7.odt]

Table supplement 2. Gradient mobile phase composition for ion-pairing LC-MS/MS for the quantification of acetyl-CoA, methyl citrate and methylmalonyl-CoA.

| Time (min) | Mobile Phase (%) |
| --- | --- |
|  | A | B |
| 1 | 100 | 0 |
| 2 | 55 | 45 |
| 7 | 50 | 50 |
| 7.5 | 5 | 95 |
| 8 | 5 | 95 |
| 8.5 | 100 | 0 |
| 10 | End |  |

A: water-methanol 07:3 (v/v) + 10mM tributylamine + 3mM acetic acid; B: pure methanol
