# Supplementary material for: Maintenance of cell wall remodeling and vesicle production are connected in Mycobacterium tuberculosis
Source: eLife. 2025 Feb 17;13:RP94982. doi: 10.7554/eLife.94982 (PMC11832169; doi:10.7554/eLife.94982)
Supplement: Supplementary file 34 [file elife-94982-supp8.zip › Supplement 8.odt]

Table supplement 3. Gradient mobile phase composition for ion-pairing LC-MS/MS for the quantification of pyruvate.

| Time (min) | Mobile Phase (%) |
| --- | --- |
|  | A | B |
| 1 | 5 | 95 |
| 6 | 95 | 5 |
| 7 | 95 | 5 |
| 8 | 5 | 95 |
| 10 | End |  |

A: pure water; B: pure methanol
