# Supplementary material for: Maintenance of cell wall remodeling and vesicle production are connected in Mycobacterium tuberculosis
Source: eLife. 2025 Feb 17;13:RP94982. doi: 10.7554/eLife.94982 (PMC11832169; doi:10.7554/eLife.94982)
Supplement: Supplementary file 35 [file elife-94982-supp9.zip › Supplement 9.odt]

Table supplement 4. Quantifier MRM transitions and collision energies used for acety-CoA, methyl citrate, methylmalonyl-CoA and pyruvate.

| Metabolite | Precursor ion [M-H]- (m/z) | Product ion (m/z) | Collision energy (eV) |
| --- | --- | --- | --- |
| Acetyl-CoA | 808.00 | 407.95 | 41 |
| Methyl citrate | 204.90 | 125.10 | 13 |
| Methylmalonyl-CoA | 432.60 | 410.50 | 10 |
| Pyruvate | 87.20 | 43.10 | 13 |
